# Clinicians’ Perspectives of a Novel Home-Based Multidisciplinary Telehealth Service for Patients with Chronic Spinal Pain

**DOI:** 10.5195/ijt.2018.6249

**Published:** 2018-12-11

**Authors:** MICHELLE A. COTTRELL, ANNE J. HILL, SHAUN P. O’LEARY, MAREE E. RAYMER, TREVOR G. RUSSELL

**Affiliations:** 1 SCHOOL OF HEALTH AND REHABILITATION SCIENCE, UNIVERSITY OF QUEENSLAND, QUEENSLAND, AUSTRALIA; 2 CENTRE FOR RESEARCH EXCELLENCE IN TELEHEALTH, UNIVERSITY OF QUEENSLAND, QUEENSLAND, AUSTRALIA; 3 PHYSIOTHERAPY DEPARTMENT, ROYAL BRISBANE & WOMEN’S HOSPITAL, QUEENSLAND, AUSTRALIA; 4 STATE-WIDE NEUROSURGICAL AND ORTHOPAEDIC PHYSIOTHERAPY SCREENING CLINIC & MULTIDISCIPLINARY SERVICE, METRO NORTH HOSPITAL & HEALTH SERVICE, QUEENSLAND, AUSTRALIA

**Keywords:** Clinician perspective, Musculoskeletal, Post-implementation, Telehealth, Telerehabilitation

## Abstract

Chronic spinal pain conditions can often be successfully managed by a non-surgical, multidisciplinary approach, however many individuals are unable to access such specialised services within their local community. A possible solution may be the delivery of care via telerehabilitation. This study aimed to evaluate clinicians’ perspectives on providing clinical care via telerehabilitation during the early implementation of a novel spinal telerehabilitation service. Eight clinicians’ were recruited, completing surveys at four separate time points. Confidence in providing treatment via telerehabilitation significantly improved with time (χ^2^(3)=16.22, p=0.001). Clinicians became significantly more accepting of telerehabilitation being a time- (χ^2^(3)=11.237, p=0.011), and cost-effective (χ^2^(3)=9.466, p=0.024) platform in which they could deliver care. Overall satisfaction was high, with technology becoming easier to use (p=0.026) and ability to establish rapport significantly improved with experience (p=0.043). Understanding clinicians’ perspectives throughout the early implementation phase of a new telerehabilitation service is a critical component in determining long-term sustainability.

Chronic spinal pain is a leading cause of pain and disability (Vos et al., 2015), affecting 16% (3.7 million) of Australians at any given time (Australian Institute of Health & Welfare, 2016). For the majority of sufferers, international guidelines recommend a non-surgical, multimodal approach that supports active self-management (Chou et al., 2007; Maher, Williams, Lin, & Latimer, 2011; National Institute for Health and Care Excellence, 2016). Unfortunately, affordable and timely access to recommended care in Australia can be limited for individuals that reside outside of metropolitan regions, often due to a lack of available primary care resources (AIHW, 2013; Department of Health, 2017). Telerehabilitation is a mode of service delivery that mitigates many of the traditional environmental barriers that prevent patients from accessing suitable healthcare (Moffatt & Eley, 2010). Furthermore, a recent systematic review reported that telerehabilitation is clinically effective for the management of a variety of chronic musculoskeletal conditions (Cottrell, Galea, O’Leary, Hill & Russell, 2016). Since 2011, the Australian government has provided financial incentives to facilitate the uptake of telerehabilitation by both primary and tertiary health care providers (MBS Online; Queensland Health, 2016). Despite this, widespread adoption of telerehabilitation into contemporary practice remains elusive (Newman, Bidargaddi, & Schrader, 2016; Zanaboni & Wootton, 2012). A lack of clinician acceptance is considered to be one reason for the poor uptake and sustainability of telehealth (Wade, Eliott, & Hiller, 2014; Whitten & Holtz, 2008; Whitten & Mackert, 2005), often stemming from barriers such as resistance to change, poor technology self-efficacy, and concerns surrounding safety and the patient-clinician therapeutic relationship (Brewster, Mountain, Wessels, Kelly, & Hawley, 2013). A key strategy to assist with the successful integration of telerehabilitation services into contemporary practice is to directly engage with frontline service providers throughout the development and implementation of the new service (Puskin, Cohen, Ferguson, Krupinski, & Spaulding, 2010).

Advanced physiotherapy-led screening clinics have been operational in hospital facilities throughout Queensland, Australia for over a decade. The primary objective of this model of care is to provide early assessment and management for patients referred to tertiary specialist surgical outpatient services with a non-urgent chronic musculoskeletal condition. Ongoing management is subsequently provided through referral to allied health professionals in a coordinated, patient-centred manner. The Spinal Physiotherapy Screening Clinic & Multidisciplinary Service (SPSC & MDS) provides services to all patients referred to the Royal Brisbane and Women’s Hospital with non-urgent spinal pain conditions. Approximately 60% of patients seen by this service reside outside the metropolitan health district and as a result many patients are unable to access appropriate multidisciplinary treatment in their local community. In response to this, a telerehabilitation clinic was established in 2017 to provide clinical services to patients in their own homes. Identical to the established SPSC & MD service, the Telehealth Clinic is comprised of a multidisciplinary team that includes physiotherapy, occupational therapy, psychology, dietetics, and pharmacy. Clinical care is provided directly into the patient’s home, using a web-based telerehabilitation platform (eHAB®, NeoRehab, Australia) that patients access via their own Internet-enabled computer device. Clinicians participating in the telerehabilitation service received a comprehensive orientation to the Telehealth Clinic, which included individualised training in the telerehabilitation platform, as well as an opportunity to provide input towards the development of the clinic’s operational processes. Clinicians were also provided off-line time (3–5 days) to become familiar with the platform and develop any resources (e.g., images/videos) that were required to deliver clinical care via telerehabilitation. These clinicians, whilst experienced in providing face-to-face clinical care within the standard SPSC & MDS model of care, had little or no experience in telerehabilitation prior to commencing this service. Therefore, this new method of service delivery provided a unique opportunity to better understand clinicians’ views towards using telerehabilitation and how they change over time. The specific aim of the study was to prospectively evaluate clinicians’ knowledge, confidence, acceptance and satisfaction towards using telerehabilitation for the management of patients with chronic spinal pain conditions throughout the initial implementation of the SPSC & MDS Telehealth Clinic.

## MATERIALS & METHODS

### STUDY DESIGN & PARTICIPANTS

This study used a repeated-measures design. Surveys were conducted at four separate time points over a six month period:

- Phase 1: Prior to the participant’s orientation into the Telehealth Clinic.
- Phase 2: Following orientation, but prior to commencing clinical activity with the Telehealth Clinic.
- Phase 3: Three months following the commencement of clinical activity within the Telehealth Clinic.
- Phase 4: Six months following the commencement of clinical activity within the Telehealth Clinic.

All clinicians directly involved with the Royal Brisbane and Women’s Hospital SPSC & MDS Telehealth Clinic during the first six months of service delivery (February – August 2017) were invited, and accepted, to take part in this study. Ethical approval to conduct this study was granted by the Royal Brisbane and Women’s Hospital (HREC/16/QRBW/440) and University of Queensland (2016001468) Human Research Ethics Committees. Written consent was provided by all participants prior to entry into the study.

### SURVEY DESIGN & DEVELOPMENT

The participant survey was developed for the specific purpose of this study, with individual items derived from two main sources. The first source included themes identified by the research team in a previously published study (Cottrell, Hill, O’Leary, Raymer, & Russell, 2017b). These themes suggested that whilst there would most likely be an improvement in patients’ access to appropriate healthcare, telerehabilitation would have significant limitations, including safety and privacy, as well as achieving clinician-patient rapport, when compared to standard face-to-face care. Results of the previous study also suggested key enablers for the successful implementation of telerehabilitation would include providing staff with training and upskilling, as well as adapting current clinical practice such that it can be delivered via telerehabilitation. The second source included theoretical constructs, such as the ‘unified theory of acceptance and use of technology’ (UTAUT), which have been developed, and validated, to explain technology acceptance and use (Davis, 1989; Venkatesh, Morris, Davis, & Davis, 2003).

The surveys consisted of the following domains: demographics, technology experience, knowledge & confidence, acceptance and satisfaction with using telerehabilitation in clinical service delivery. Demographic and technology experience domains were completed only at the initial time point (Phase 1). Knowledge & confidence, and acceptance domains were repeated at all four time points, whilst the satisfaction domain was made available for only the final two time points (Phase 3 and 4). Participants scored individual items using a continuous 100mm visual analogue scale (VAS), where 0 represented ‘complete disagreement’ (acceptance and satisfaction domains) or ‘no knowledge/confidence at all’ (knowledge & confidence domain), to 100 which represented ‘complete agreement’ or ‘extremely knowledgeable/confident’. The survey was pilot-tested by a small group (n = 3) of clinicians associated with the SPSC & MDS, but who were not directly involved with the Telehealth Clinic. Feedback that was provided resulted in minor word, style and formatting changes.

### DATA ANALYSIS

Data was analysed using SPSS software Version 24 (IBM, Chicago, USA). Demographic and technology experience data was recorded categorically, collated and presented using descriptive statistics. Responses for individual items for the remaining domains were collated and medians calculated for each time point, whilst median differences were calculated between each time point (Mdn diff). Non-parametric Friedman tests were undertaken to determine any significant change in score response over the four time points for individual items addressing the knowledge & confidence, and acceptance domains. In the event of statistical significance (p ≤ 0.05), post-hoc pairwise comparisons were performed with a Bonferroni correction for multiple comparisons. Differences in individual item responses for the satisfaction domain were assessed using Wilcoxon signed-rank tests where the threshold for significance was set at p ≤ 0.05.

## RESULTS

### DEMOGRAPHICS & TECHNOLOGY EXPERIENCE

A total of eight participants were recruited to this study. Due to staff changes, two participants were unable to complete all four time points, and therefore only six participants were included in the analysis. Participants represented all healthcare professions of the SPSC & MDS Telehealth Clinic. Participants reported approximately ten years clinical experience within their nominated profession, and up to two years’ clinical experience with the SPSC & MD service as treating clinicians in the conventional face-to-face delivery of care. Participants reported frequent personal use of a variety of different technology platforms, but had limited experience in using technology as part of clinical practice. Only one participant reported minimal (i.e., one episode of care) telerehabilitation experience prior to commencing with the SPSC & MDS Telehealth Clinic.

### KNOWLEDGE & CONFIDENCE

**Figure 1:**
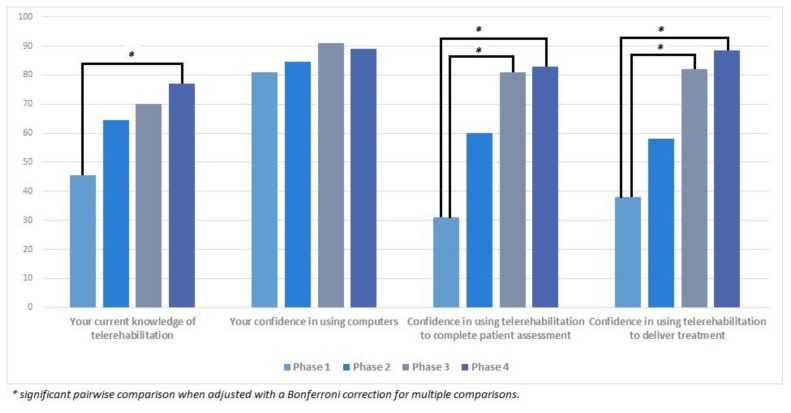
Participants’ change in knowledge and confidence towards telerehabilitation.

Results for the participants’ change in knowledge and confidence over time are presented in Figure 1. Knowledge of telerehabilitation significantly increased over time (χ^2^(3) = 14.6, p = 0.002). Post-hoc analysis identified that statistically significant differences in knowledge occurred between Phase 1 and 4 (Mdn diff = 28.5, p = 0.001), but not between any other time points. Confidence in using computers slightly increased over time, however this difference was not statistically significant (χ^2^(3) = 6.052, p = 0.109). Participants’ confidence in using telerehabilitation to complete a patient assessment (χ^2^(3) = 12.2, p = 0.007) significantly improved with experience. Post-hoc analysis revealed that improvements were statistically significant following three- (Mdn diff = 45, p = 0.022) and six months (Mdn diff = 42, p = 0.01) of telerehabilitation experience. Similar results were seen for confidence in delivering treatment via telerehabilitation (χ^2^(3) = 16.22, p = 0.001), where post-hoc analysis revealed that statistically significant improvements also took place after three- (Mdn diff = 34.5, p = 0.022), and six-months (Mdn diff = 43.5, p = 0.001).

### ACCEPTANCE

**Figure 2:**
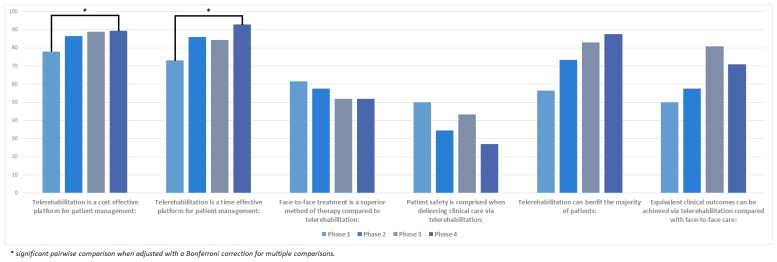
Participants’ change in acceptance towards telerehabilitation.

Participants’ change in acceptance towards using telerehabilitation are presented in Figure 2. Following six months of telerehabilitation experience, participants’ attitudes towards telerehabilitation as being both a time- (χ^2^(3) = 11.237, p = 0.011) and cost-effective (χ^2^(3) = 9.466, p = 0.024) platform to deliver clinical care significantly improved. Over time, participants started to disagree that face-to-face treatment is a superior method of service delivery when compared to telerehabilitation, however this change was not significant (χ^2^(3) = 5.948, p = 0.114). Concerns surrounding the compromise of patient safety when using telerehabilitation continued to decrease over time (Mdn = 27), however this change also did not reach significance (χ^2^(3) = 6.763, p = 0.08). Acceptance of telerehabilitation being of clinical benefit to the majority of patients increased between each time point (χ^2^(3) = 6.310, p = 0.097), with the largest change (Mdn diff = 19.5) seen following the participants’ orientation to the Telehealth Clinic. Finally, participants considered that clinical outcomes achieved via telerehabilitation would be equivalent to face-to-face care, and whilst not significant (χ^2^(3) = 4.358, p = 0.225), this level of acceptance did increase over time.

### SATISFACTION

All participants were overwhelmingly satisfied with providing clinical care via telerehabilitation after just three months of clinical experience, as evident in Table 1. This level of satisfaction was either maintained, or increased, at six months, however due to the high initial satisfaction, statistically significance improvements in satisfaction was only reached for items relating to technology ease-of–use and patient-clinician therapeutic rapport.

## DISCUSSION

The results of this study demonstrate that clinicians’ confidence, acceptance and satisfaction towards using telerehabilitation were positive throughout the early implementation of the SPSC & MDS Telehealth Clinic. Interestingly, the results of this study were primarily in contrast to the themes identified in pre-implementation work recently published by the research team (Cottrell et al., 2017b), where there were apparent barriers and hesitation towards the uptake of telerehabilitation for this service. With only one participant (out of 26) in the previous study having any telehealth experience, the results of the present study signify that direct clinical experience may be what is required to achieve positive perceptions towards telerehabilitation. As a result, when considering the adoption of telerehabilitation into standard clinical practice, understanding the experiences and acceptance of front-line clinicians’ involved in providing services via telerehabilitation should form an integral component of the formal evaluation process (Whitten & Mackert, 2005).

Despite a high level of confidence in the general use of computers, clinicians initially demonstrated limited confidence and knowledge in the use of telerehabilitation, which can be explained by only one participant having previous telehealth experience. This confidence increased, albeit not significantly, following the clinicians’ orientation to the Telehealth Clinic. These results support previous literature in suggesting that specific, hands-on training acts as a key facilitator of acceptance towards technology (Brewster et al., 2013; Broens et al., 2007), and therefore adequate time and resources should be allocated for training when implementing a telerehabilitation service. Managing the change process when implementing telerehabilitation can also be exceptionally difficult and time-consuming, as it is often a substitute for existing work practices (Giordano, Clark, & Goodwin, 2011). Subsequently, technology choice also plays an imperative role in the acceptance of telerehabilitation, as theoretical constructs such as the Technology Acceptance Model suggest that information technology options that are perceived as easy-to-use and user-friendly are more likely to be adopted (Davis, 1989). Technology must also be chosen based upon the needs and objectives of the individual service, rather than attempting to retrofit a service to equipment that may be cheaper or already available (Broens et al., 2007; Giordano et al., 2011; Hines, Lincoln, Ramsden, Martinovich, & Fairweather, 2015). Regardless of the chosen telerehabilitation platform, operational resources should also be allocated to providing ongoing skills development and technical support beyond the initial implementation phase, as a way of sustaining positive attitudes towards telerehabilitation (Brewster et al., 2013; Hines et al., 2015; Odeh, Kayyali, Gebara, & Philip, 2014).

Even prior to any telerehabilitation experience, there was a strong agreement amongst participants that telerehabilitation was a cost-effective medium in which patients were able to access care. This belief may be derived from the fact that patients are able to receive treatment directly into their home, negating the need for travel and subsequently removing the majority of costs traditionally incurred by patients when trying to access healthcare (Wade, Karnon, Elshaug, & Hiller, 2010). Subsequently, as patients are also required to have access to their own Internet-enabled computer device, it can be postulated that there is a negligible financial impact on the patient when receiving care from the SPSC & MDS Telehealth Clinic. Further economic analyses, from both a societal and health service perspective, of the service under study are currently taking place to confirm this hypothesis.

Participants did not consider patient safety to be compromised when engaging in home telerehabilitation, despite the literature consistently reporting the potential risk to patient safety as a barrier to the successful implementation of telehealth services (Brewster et al., 2013; Mair et al., 2007; Mair et al., 2005). Breaches of privacy and confidentiality, which have again been reported as a barrier to implementing home telehealth (Kruse et al., 2016; Newman et al., 2016), were also not considered to be of major concern. The disparity between previous literature and current findings may be due to the majority of published literature involving the perceptions and experiences of nurses’ towards telehealth for the management of patients with chronic cardiac and pulmonary conditions (Brewster et al., 2013; Giordano et al., 2011). These services which were telemonitoring in nature, were often implemented as a substitute for traditional in-home nursing services, and frequently relied on patients being able to navigate specialised equipment to monitor physiological parameters (e.g., blood sugar levels, blood pressure, etc.). This suggests again that the barriers and facilitators towards clinicians’ acceptance and satisfaction of a telerehabilitation service may be dependent on the healthcare professions involved as well as how the specific healthcare services are being provided (e.g., via telemonitoring equipment, real-time consultations), and therefore results should not be generalised across other telehealth service models.

Overall clinicians’ had a high level of satisfaction with what could be achieved via telerehabilitation. This satisfaction did not diminish with experience, thus providing support for the long-term sustainability of the service under investigation (Wade et al., 2014). Whilst the high level of satisfaction with patient-clinician rapport may have been facilitated by the audio and visual quality achieved by the telerehabilitation platform used in this study, home telerehabilitation also provides a level of contextual relevance that cannot be replicated within the clinic environment. Clinicians, in particular physiotherapists and occupational therapists, have the ability to observe how patients’ function within their own environment with a vehicle to provide immediate feedback that is specific to the individual patient. Therefore, this contextual relevance may not only have the ability to strengthen the patient-clinician relationship, but also provide advantages to clinical care that are unable to be achieved in the traditional face-to-face clinic environment.

There were strengths and limitations of this study. Limitations include a small sample size, which was of a multidisciplinary nature and derived from one specific telerehabilitation service. Nonetheless, this is one of the first studies to evaluate clinicians’ perceptions towards delivering treatment via telerehabilitation for this patient cohort as part of a ‘real-world’ pragmatic healthcare service. The repeated-measures design also made it possible to monitor these perceptions and how they change with time and experience, thus providing more information about the magnitude and timing of these changes. This study is just one in a series of studies that evaluates the development and implementation of the SPSC & MDS Telehealth Clinic (Cottrell, Hill, O’Leary, Raymer, & Russell, 2017a; Cottrell et al., 2017b). Studies currently underway are evaluating both the clinical and economic (from both patient and healthcare sector perspectives) outcomes of telerehabilitation for the management of patients with chronic spinal pain conditions when compared to accessing in-person services within the local community. Future studies will also be undertaken to evaluate the attitudes of patients receiving care from the SPSC & MDS Telehealth Clinic. Whilst unable to generalise, patient satisfaction has repeatedly been shown to be high when receiving management for a variety of musculoskeletal conditions via telerehabilitation (Eriksson, Lindström, & Ekenberg, 2011; Moffet et al., 2017), and that patients’ acceptance and satisfaction often exceeds that of the clinicians treating them (Ward, Burns, Theodoros, & Russell, 2013).

This study evaluated clinicians’ confidence, acceptance and satisfaction of providing clinical care via telerehabilitation for the non-surgical management of patients with chronic spinal pain conditions, and how these perspectives changed over time. Overall, results were positive but also suggested that acceptance towards telerehabilitation can be significantly improved with both formal hands-on training and direct clinical experience. Future studies should be undertaken to formally evaluate patients’ perceptions towards telerehabilitation for the treatment of their musculoskeletal condition, as well as compare the perceptions of clinicians’ from different services and jurisdictions.

## Figures and Tables

**Table 1:** Participants’ Satisfaction towards Telerehabilitation Following Three- and Six-months Experience

#	Item	Phase 3 (median/100)	Phase 4 (median/100)	Wilcoxon signed-rank test (p ≤ 0.05)
**1**	I find the telerehabilitation system easy to use:	88	94	**0.026**
**2**	I am satisfied with the audio quality of the telerehabilitation system:	80	85	0.249
**3**	I am satisfied with the visual quality of the telerehabilitation system:	74	83	0.225
**4**	I am not satisfied with the level of clinician-patient rapport that I can achieve with telerehabilitation:^a^	24	9	**0.043**
**5**	I feel that telerehabilitation negatively impacts on patient privacy:^a^	23	20	0.893
**6**	I feel that patients can easily follow my instructions during a telerehabilitation appointment:	80	79	0.917
**7**	I feel that I am unable to competently assess/treat patients via telerehabilitation to the extent that I believe is required.^a^	15	10	0.172
**8**	I don’t feel comfortable using the telerehabilitation equipment:^a^	8	7	0.273
**9**	Overall, I am satisfied with the clinical outcomes I can achieve via telerehabilitation:	84	94	0.08
**10**	Overall, I am satisfied with the level of service telerehabilitation allows me to provide patients:	93	92	0.345

*Note*.

a Negatively-keyed item responses.
